# Surgical Suggestions

**Published:** 1907-05

**Authors:** 


### SURGICAL SUGGESTIONS.

---

In the Bier treatment, the cup will stay in place without other assistance if zinc oxide salve or vaselin is applied to the skin. Even better than this is a piece of smooth rubber tissue which protects the skin from irritation by the pus.

In the case of gastric disease of doubtful diagnosis, progressive loss of weight is the most important sign in determining the probability of carcinoma.—American Journal of Surgery.

---

By adding a one-fourth per cent., or stronger, solution of boracic acid to Burow's solution (aluminum acetat), the latter clears at once, if cloudy, and remains permanently free from turbidity or precipitation.—American Journal of Surgery.

---

The sudden acute onset of abdominal pain with tenderness over the appendix region but with rigidity of the right rectus low down, is very suggestive of acute salpingitis. The diagnosis is further confirmed if there is high temperature and extremely high leucocyte count (20,000-40,000; polynuclears, 80-90 per cent), even though viginal examination be negative.—American Journal of Surgery.

